# 

**Published:** 1912-11-16

**Authors:** 


### BUREAU OF INFORMATION.

### Rules for Correspondents.

1. Every letter, on whatever subject, **must be accompanied by the coupon** to be cut from the back cover (inside page) of The Hospital, current issue, and must contain the name and address of the correspondent with pseudonym for publication if desired.
2. Replies by post cannot be given, save under exceptional circumstances at the Editor's discretion.
3. Letters from Approved Homes in reply to special needs published in the Bureau should state terms and full particulars, and be sent prepaid under cover to the Editor of the Bureau with coupon and name enclosed for identification.
4. Proprietors of Homes which have not yet been entered on the List of Approved Homes, but have spare accommodation likely to suit special needs, are invited to write for an application form for registration. The fee for registration, which includes two announcements of the Home in the Bureau, is 5s.
5. Special needs of every description relating to those who are sick or in difficulties are made known in these columns **free of charge.**
6. All communications to be addressed to The Editor of The Hospital, 28 Southampton Street, Strand, London, W.C., and marked "Bureau of Information."

### Nursing Homes Wanted.

**For Aged Lady.**—The patient is advanced in years, and the friends want to find a kind Home where she could be received for 15s. a week. Prepaid replies to R. E. M. (133a).

### Needs and Helpers.

**Home for Defective Boy.**—The Metropolitan Asylums Board has made a praiseworthy attempt to deal with the immense demand for institutional accommodation for the class of case you describe, but the ground is by no means covered. Write to the Secretary of the Children's Committee of the Metropolitan Asylums Board, Victoria Embankment, E.C. Your case may be eligible for admission to their Bridge Industrial Home, Witham, Essex. If the Bill dealing with the feeble-minded becomes law this session, there will no longer be so much difficulty in providing for children who need special training, and the parents might be wise to wait events.—Miss F., Hampstead.

**Assisted Passages to New Zealand.**—The family of which you send particulars appear to represent the high-water mark of emigration. There are so many societies dealing with the problem you experience of finding the passage money that they have formed a voluntary union and you should apply to headquarters, The Associated Societies' Voluntary Committee, Royal Colonial Institute, Northumberland Avenue, London. It will probably be more satisfactory for the man to go out first, as largely reduced passages are available for families joining their relatives.—Mrs. H., Bexhill.

**Sanatorium Treatment at Five Shillings a Week**—We regret to say that there is no possibility of the girl you mention as about to be discharged from Brompton Hospital being received in any sanatorium for this sum. We have endeavoured without success to procure treatment for similar cases at twice the amount. We can only suggest that she should apply for admission to be received into the Poor-Law infirmary on leaving Brompton, where at least she would have the benefit of admirable nursing and good food. If it were an advanced or hopeless case free treatment might be procurable, but we gather there is hope of cure in good surroundings. This makes it the more distressing. We trust one of our readers may be able to offer a suggestion.—Sister S.W.

**Hand-Propelled Tricycle.**—Wanted for naval stoker, paralysed when on duty, a hand chair or tricycle in which he could move himself about. The lower limbs are helpless and medical adviser recommends exercise as best aid to recovery. Has any reader a machine of this description for sale second-hand or for hire? Replies forwarded to Captain (134x).

**Blind and Deaf.**—Alas, we must not, I fear, aim at the thirty guinea donation. But the two guinea subscription ought to be quite within our power, and before Christmas too. We will make ourselves responsible for one guinea yearly if you and your friends will promise the other. Set them to work and explain to them that every shilling they promise will count for two immediately and in course of time will bring you in four.—D. B., Maidstone.

### Training and Employment.

**Sewing-Maid in Institution.**—We are pleased to make it known that you can recommend a good sewing-maid who has been trained as a dressmaker and wishes to enter an institution where the conditions of work would be more healthy than in a town workroom. We understand she is willing to begin at a low wage. Prepaid letters forwarded to F. H.

**Nursery Nurses.**—The fees are not low in any case for this kind of training, as services are not of value during training as in nursing, and the expenses are heavy. Write to Sesame House, 43a Acacia Road, St. John's Wood, N.W.—Eva.

**How to Inaugurate a Nursing Home.**—By all means have a "view day" for any who are at all likely to be interested in your new Home. In sending invitations be careful to mention morning hours as well as afternoon; say from 11 to 1 and from 3 to 5. You can offer a cup of tea to the later comers. You will do well to remember that officials such as the postmaster and the stationmaster, as well as the leading chemists, house agents, etc., are likely to be asked for such accommodation as you offer, and they will appreciate the courtesy of an invitation to inspect your premises. The ministers of the Free Churches should be invited with their wives, as well as the clergy and any local residents of whom you have some acquaintance, and these may also be asked to bring friends. Nothing, however, is to be gained by sending notices to entire strangers.—E. M. E.

**Partner Required for High-Class Nursing Home in London.**—A fully-trained nurse with maternity training, who has some connection and a little capital, is wanted to join principal of fully-equipped Home on our List, which has been long-established and is doing well. "Churchwoman who would have an interest in the spiritual side of her work as well as in the other" desired. Prepaid letters forwarded to ZZZ, who will furnish full particulars.

### Nursing in the Empire and Abroad.

**Municipal Nursing in Brussels.**—The papers you enclose are from the Municipal Hospital Service in Brussels, and we think you might rely on everything being perfectly straightforward in connection with the post offered. They evidently want to introduce English methods into their wards, and the work, though probably rather uphill at first, would be interesting and valuable. The post which answers to our sister is that of "surveillante-adjointe," commencing at 1,600 francs a year and advancing to 2,400. You had better find out whether this includes board and lodging. We think fluent conversational French would be indispensable to success. For particulars relating to German hospitals see the illuminating series of articles now appearing in our columns from the pen of Sir Henry Burdett, K.C.B., K.C.V.O., who has recently made an exhaustive survey of the German hospital and nursing system. We imagine English nurses would not be received with any cordiality by German doctors.—Moyra.

**Nairobi Nursing Association.**—Nurses command £3 to £5 per week. All applications from nurses wishing to come out should be sent to The Secretary of the East Africa Nursing Association, Nairobi.—Gertrude B.

**Nurses Wanted in Alberta.**—A favourable opportunity offers for nurses desiring to emigrate to Canada. The Scottish Nursing Home and Association, Calgary, Alberta, has proved so successful that immediate expansion is called for, and additional nurses, especially if they be of Scotch nationality, will be warmly welcomed. Full information can be obtained from Miss E. S. Paterson, 11 Regent Square, Lenzie, N.B. Nurses joining the Scottish Association bind themselves for six month only, after which they are at liberty to take any post which offers or to take up private nursing.

**Nursing in the Malay States.**—All the nursing associations and hospitals in these States obtain their nurses through the medium of the Colonial Nursing Association, Imperial Institute, London, S.W., to whom you should apply if you wish to be sent out to this part of the world. You will be expected on first joining the Association to go wherever you are sent, but no doubt your wishes would be respected as far as possible in the choice of a locality.—Miriam.

### Letter-Box.

Miss Hannaford.—We congratulate you on the addition of a well-equipped surgical theatre to your Home at 14 Whittingstall Road, Fulham.

Mr. L. G.; Matron C. M. H.—You will see by our rules that we are unable to introduce any but Homes on our Approved List to the patients whose wants are made known in our columns.

L. N., Port Tewfik.—We are doing our utmost to forward your wishes, and are in communication with several sources through which the desired result may be obtained.

Dr. Scott, Kuching; Dr. Fleming, Salisbury, Rhodesia; W. B. Heard, Sofia; C. White, Yokohama.—Greatly indebted to you for valuable particulars received.

**Communications have been received and attended to from:—**

The Rev. J. M. A., Co. Galway; Miss A. S., Penn, Staffs.; Miss W. A., Co. Dublin; Mr. E. M. B., Northampton; Mrs. G., Chelmsford; Miss J. G. N., Stratford-on-Avon; Miss S., Dulwich; Miss C. R., London; Miss P., Hove; Miss M. D., Highgate; Miss B. P., Newark; the Rev. J. H., Merioneth; the Rev. E. A. S. L., Luton; Miss R., Chiswick; Nurse J., Middlesbrough; the Rev. L. A., Chippenham; Deaconess G., Cranleigh; Miss A. S., Halifax; Mrs. H., Orlestone; Miss H. M. B., Teath; Miss P., Tunbridge Wells.

*** All who are sick or in difficulties have the opportunity of making their wants known free of charge under a pseudonym in the Bureau, and can thus be brought under the notice of those willing and able to render assistance. A large number of the Nursing Homes on the Approved List have generously signified their willingness to receive patients in poor circumstances recommended to them through the Bureau at greatly reduced terms. No charge is made on either side for the introduction of patients, and the advantages afforded by this free intercommunication between patients and Homes of tried efficiency should be noted by all members of the medical profession.

### Buildings Contemplated and in Progress.

Birmingham.—Messrs. C. Whitwell and Son are architects for homes for epileptic and feeble-minded children and adults and other buildings at Monyhull Colony, King's Heath. A deposit of £25 is asked for particulars of the contract.

Epsom.—The ground wall of the new county asylum at Epsom has been commenced. Accommodation for 2,000 patients will be provided.

Exeter.—The Exeter Guardians have applied for sanction to a £7,000 loan for a children's home adjoining the workhouse.

Gravesend.—Mr. E. J. Bennett, A.R.I.B.A., is architect for additions at Gravesend Workhouse. Tenders are being considered by the Guardians.

Middlesex.—Mr. Rowland Plumbe, F.R.I.B.A., is architect for alterations at Napsbury Asylum estimated to cost £6,461. The purpose of the works is to increase the administrative accommodation.

Newtown.—The Montgomeryshire Sanatorium Committee has approved the erection of a consumptive hospital close to Newtown.

Paignton.—Alterations and additions, including new wards, are projected at Paignton Isolation Hospital. Particulars are being forwarded to the Local Government Board for approval.

Rochford.—Mr. Walter J. Wood, of Southend-on-Sea, is architect for an aged and infirm inmates' block at Rochford Union Workhouse. Tenders are invited for the works and the contract contains a guarantee clause.

Sheffield.—The Ecclesall Bierlow Guardians invite architects to submit competitive designs for temporary hospital accommodation to include twenty-four beds. Financial liability is not undertaken for rejected plans and no mention is made of a professional assessor.

Shildon.—Tenders of £3,137 and £2,977 have been accepted respectively for isolation hospital additions at Tindale Crescent and Helmington Row.

Streatham.—Messrs. E. T. and E. S. Hall are architects for the Queen Alexandra Wing at the Crown Lane Incurable Home and Hospital. The work is expected to cost close on £10,000.

Wakefield.—The West Riding County Council (Yorks) has resolved to apply for sanction to a loan of £5,000 for the purchase of a sanatorium site.

Warminster.—Plans for an isolation hospital by Mr. C. H. Lawton are to be forwarded to the Local Government Board for approval. The estimated cost of the buildings is £3,600.

West Harton.—Mr. J. H. Morton, of Newcastle-upon-Tyne, is architect for extensions at West Harton Workhouse. Tenders are invited and quantities may be had on deposit of £2 2s.

Wimbledon.—An additional ward is projected for Wimbledon Isolation Hospital from details prepared by the Council's surveyor. The work is estimated to cost about £4,000.

Winsley.—Winsley Sanatorium, Bradford-on-Avon, is to be extended at a cost of £3,993.

### The Editor's Letter-Box.

### Nelson Hospital.

WITH reference to the plan of the Nelson Hospital published in our issue of September 21, Mr. R. A. Hinds, F.R.I.B.A., requests us to state that the building was designed by Mr. Francis Hatch, Lic.R.I.B.A., and carried out by Mr. Hinds. We must adhere to our criticisms, which were mainly administrative, and have to do mostly with considerations of hygiene, comfort, and reasonable privacy.—ED. THE HOSPITAL.

### Gifts and Bequests.

THE Chelsea Hospital for Women has received £100 from Mr. Basil C. Fothergill towards the rebuilding of the hospital and its nurses' home.

KING EDWARD'S HOSPITAL FUND for London has received £500, the current year's instalment of the £2,500 voted by the Corporation of the City of London.

MR. EDWARD GRAHAM WOOD, of Manchester and Salford, has presented a cheque for £1,000 to endow a bed at St. Mary's Hospital for Women and Children, Manchester. This is the fourth bed which he has endowed in the district.

MR. WILLIAM RIGBY, of Leigh, Lancashire, retired farmer, has bequeathed £1,000 to the maintenance fund of the Leigh Infirmary for a "William Rigby" bed, and the residue upon trust for investment to pay the income to the same fund.

MR. THOMAS BARTLETT, of Liverpool, has left sufficient sums to endow a bed in each of five Liverpool hospitals in memory of the members of his family, and £20,000 to the Home for Epileptics, Maghull, for the erection of a home to be called the Thomas Bartlett Home.

THE Municipal Council of Vienna, in recognition of their recent visit to London, have sent to the Lord Mayor 5,000 kronen (£206 3s. 8d.) for any charitable object in the City of which he approves, and for the personal dispensation of which he will be responsible. This sum Sir Thomas Crosby has handed over to the Special Appeal Fund on behalf of St. Bartholomew's Hospital.

MRS. JAMES, the widow of the late Mr. William D. James, of West Dean Park, Chichester, has handed over to the Chichester General Infirmary a cheque for £1,453, which represented the amount collected by "the personal friends of the late Mr. Willie James," to be devoted, primarily, to the erection of balconies for men, women, and children, and the furnishing of the operating theatre, as a memorial to him. The board very gratefully accepted the gift, which will be added to the fund for the reconstruction of the infirmary.

THE Tunbridge Wells General Hospital has been bequeathed the sum of £100 by the will of the late Mrs. Ewart, 6 Mount Ephraim, Tunbridge Wells. The sum of £30 has also been received from the League of Mercy and £82 received from the Committee of the Tunbridge Wells Hospital Saturday Fund.

### A SURGEON'S BEQUESTS.

MR. CLINTON THOMAS DENT, F.R.C.S., chief surgeon to the Metropolitan Police, formerly Hunterian Professor at the Royal College of Surgeons, a former president of the Alpine Club, who left estate of the gross value of £117,661, bequeathed £1,500 to the Belgrave Hospital for Children, whom failing to St. George's Hospital, and his medical and surgical works to the Students' Library of St. George's Hospital.

### Appointments.

MR. D. A. MACPHERSON has been appointed fifth assistant medical officer at Long Grove Mental Hospital.

DR. C. S. BREBNER, of Widnes, has been appointed medical officer of health by the Chiswick Urban District Council.

DR. D. C. KIRKHOPE, assistant medical officer of health at Leyton, has been appointed medical officer of health and school medical officer at Tottenham.

THE Gloucester Sanatorium Committee have appointed Dr. John Guy tuberculosis officer under the Insurance Act. After graduating M.D. at Glasgow University and taking the Cambridge D.P.H. Dr. Guy engaged in general practice, and then became medical superintendent of the Bridge of Weir Sanatorium, Glasgow.

THE following appointments have been made at Manchester University :—Lecturer in Obstetrics and Gynæcology, Mr. W. E. Fothergill, M.D., in addition to Mr. A. W. W. Lea, M.D., who already holds the position of Lecturer in these subjects; Assistant Lecturer in the same subjects, Mr. W. Fletcher Shaw, M.D. Mr. W. K. Walls, M.B., M.R.C.S., L.R.C.P., has been appointed Lecturer in Clinical Obstetrics and Gynæcology.

Mr. A. E. Boycott, B.Sc., M.A., M.D., B.Ch., has been appointed to the Chair of Pathology, vacant through the resignation of Professor Lorrain Smith on his appointment to the Chair of Pathology in the University of Edinburgh. Dr. Boycott, a classical scholar of Oriel College, Oxford, became Fraser Research Scholar in 1899, graduated in medicine in the University of Oxford in 1902, and took the M.D. in 1904. In 1903 he was elected a Fellow of Brasenose College in recognition of his researches in pathology. After a distinguished career as a student at St. Thomas's Hospital and a wide experience of teaching in connection with pathology, he held the position of Gordon Lecturer in Pathology at Guy's Hospital, and afterwards was for two years on the staff of the Lister Institute of Preventive Medicine. Dr. Boycott then returned to Guy's Hospital as Lecturer in Pathology. His best known pathological researches are in the subject of Ankylostomiasis in Cornwall and the Prevention of Caisson Disease.

Mr. John Burton, chief attendant at the West Riding Mental Hospital at Menston, has resigned after twenty-four years' service. He will be succeeded by Mr. W. W. Sampson, who has been deputy chief attendant for twenty-one years.

### Hospital Needs.

An adjourned sessional meeting will be held at the Royal Sanitary Institute, 90 Buckingham Palace Road, London, S.W., on Tuesday, November 19, at 8 p.m. for the purpose of continuing the discussion on "The Report of the Departmental Committee on Intercepting Traps in House Drains" opened by Mr. H. Percy Boulnois, M.Inst.C.E.

Dr. Dudfield will read a paper entitled "Still-births in Relation to Infantile Mortality" at a meeting of the Royal Statistical Society to be held on Tuesday next, the 19th instant, at the Rooms of the Royal Society of Arts, John Street, Adelphi, W.C. The paper will deal with the Report of a Special Committee appointed by the Council of the Royal Statistical Society to inquire into the systems adopted in different countries for the registration of births (including still-births) and deaths, with reference to infantile mortality.

